# Evolution of Mood Symptomatology Through the COVID-19 Pandemic: Findings From the CovidSense Longitudinal Study

**DOI:** 10.7759/cureus.29876

**Published:** 2022-10-03

**Authors:** Nidal Moukaddam, Vishwanath Saragadam, Mahsan Abbasi, Matt Barnett, Anil Kumar Vadathya, Ashok Veeraraghavan, Ashutosh Sabharwal

**Affiliations:** 1 Menninger Department of Psychiatry, Baylor College of Medicine, Houston, USA; 2 Electrical & Computer Engineering, Rice University, Houston, USA; 3 Electrical & Computer Sciences, Rice University, Houston, USA

**Keywords:** longitudinal, mental health, covid-19, anxiety, stress, depression

## Abstract

Background

The severe acute respiratory syndrome coronavirus 2 global pandemic, with its associated coronavirus disease 2019 (COVID-19) illness, has led to significant mental, physical, social, and economic hardships. Physical distancing, isolation, and fear of illness have significantly affected the mental health of people worldwide. Several studies have documented the cross-sectional elevated prevalence of mental anguish, but due to the sudden nature of the pandemic, very few longitudinal studies have been reported, especially covering the first phase of the pandemic. CovidSense, a longitudinal adaptive study, was initiated to answer some key questions: how did the pandemic and related social and economic conditions affect depression, which groups showed more vulnerability, and what protective factors emerged as the pandemic unfolded?

Methodology

CovidSense was deployed from April to December 2020. The adaptive design enabled adaption to fluctuating demographics/health status. Participants were regularly queried via SMS messages about their mental health, physical health, and life circumstances. The study included 1,190 participants who answered a total of 18,783 survey panels. This was a prospective longitudinal cohort study following adult participants in the general population through the COVID-19 pandemic. The participant cohort reported self-assessed measures ranging from subjective mood ratings and substance use to validated questionnaires, such as the Quick Inventory of Depressive Symptoms (QIDS) and Cognitive and Affective Mindfulness Scale-Revised (CAMS-R).

Results

Participants with pre-existing physical (especially pulmonary) or mental conditions had overall higher levels of depression, as measured by the QIDS and self-reported mood. Participants with pre-existing conditions also showed increased vulnerability to the stress caused by watching the news and the increase in COVID-19 cases. Younger participants (aged 18-25 years) were more affected than older groups. People with severe levels of depression had the most variation in QIDS scores, whereas individuals with none to low depressive scores had the most variability in self-reported mood fluctuations.

Conclusions

The effects of pandemic-related chronic stress were predominant in young adults and individuals with pre-existing mental and medical conditions regardless of whether they had acquired COVID-19 or not. These results point to the possibility of allocating preventive as well as treatment resources based on vulnerability.

## Introduction

Elevated prevalence of psychological distress, including depression, anxiety, and behavioral correlates (sleep, substance use), was noted during the pandemic [1], more so in the general public than the medical staff [2]. Hundreds of published papers have documented increases in anxiety and depression, but few have been longitudinal, given the abrupt nature of the pandemic. A three-month survey of a non-representative UK sample showed at least four trajectories for depression symptoms and five for anxiety symptoms, and a third of the sample did not follow any of the trajectories [3]. The presence of multiple trajectories may explain the discrepant data reported in other studies, e.g., rates of depression and anxiety were not thought to increase in the first few weeks of lockdown in Ireland [4], but later caught up with reported elevations in other countries. Risk factors from the larger literature indicate that younger age, lower resilience, higher loneliness, and higher somatic problems were correlated with increases in depression and anxiety [5,6]. Having a higher resilience allowed for better coping with the pandemic restrictions, though the exact mechanism by which resilience helps is not fully elucidated [7]. Rates increased starting the first lockdown week in multiple countries, which makes sense given that life stressors can trigger depression in predisposed individuals [8,9].

Children and adolescents were reported to be at high risk for anxiety and depression compared to adults during the pandemic according to a meta-analysis [1]. In this group, excessive internet and gaming use accompanied these symptoms [10,11]. The decline in mental wellness can be explained by reduced social interactions, difficult service availability, large-scale layoffs, reduced salaries, and the constant fear of contracting the disease.

Existing literature on the effect of coronavirus disease 2019 (COVID-19) on mental health largely focuses on narrow periods in time, or a single country, making it difficult to understand the temporal dynamics of the pandemic. This is especially important now that the after-effects are being felt with sharp increases in mental health crises, depression, and anxiety globally even though the restrictions have largely ended. This precludes a clear understanding of individual responses to the prolonged pandemic period. Some studies have identified different vulnerable populations to the mental health risk of the pandemic. While most individuals generally have difficulty dealing with societal disruptions, the pandemic’s impact on individuals with pre-existing mental illness is understood to be significant as major life events often precipitate relapse of mental illness [9,12]. In this adaptive, longitudinal study, we examine the progression of mood, depression, anxiety, and substance use in light of socio-economic parameters during the COVID-19 pandemic. Using an adaptive design, we followed a cohort of 1,190 individuals from 65 countries and tracked the progression of adaptive self-reported parameters. The main hypothesis of this study was that each individual’s reaction to the pandemic would be affected by personal traits, such as mindfulness ability, personality characteristics, pre-existing health conditions, as well as external conditions such as news events and local and global pandemic dynamics. The main hypotheses were as follows: individuals in the age group of 25-40, participants with pre-existing conditions, and participants who were directly affected by the disease by either contracting it or being a caretaker would be more affected by the pandemic.

## Materials and methods

CovidSense, an adaptive longitudinal study, was launched on April 6, 2020, across 65 countries with the goal of gathering data on the mental well-being of individuals during the COVID-19 pandemic. The recruitment was mostly done through Facebook and YouTube, and some participants were recruited by word-of-mouth. The questionnaires were shared at weekly and biweekly intervals from April 6 to December 31, 2020, with panels adapted to each participant’s prior responses.

**Figure 1:**
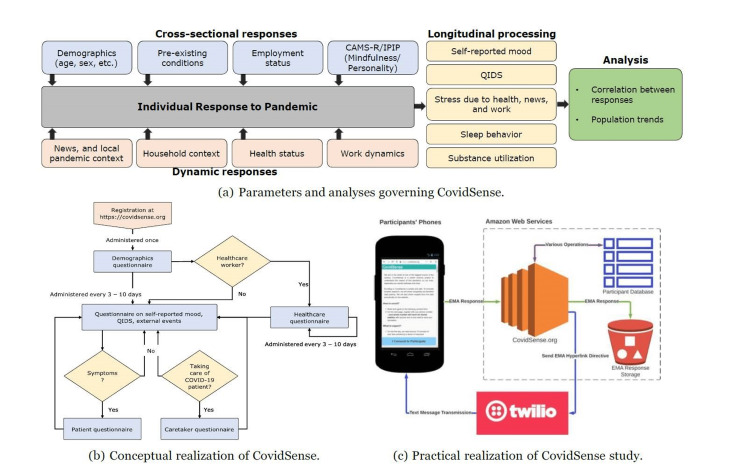
CovidSense overview. (a) CovidSense was targeted at understanding how people reacted to the ongoing COVID-19 pandemic. The study was modeled to measure cross-sectional responses, such as demographics and personality questions, and dynamic responses, such as a periodic questionnaire asking each participant’s reactions to external events. The questionnaire delivery system was designed based on the flowchart in (b) and realized as a cellphone message delivery-based system in (c). COVID-19: coronavirus disease 2019; QIDS: Quick Inventory of Depressive Symptomatology; CAMS-R: Cognitive and Affective Mindfulness Scale-Revised; IPIP: International Personality Item Pool

**Figure 2:**
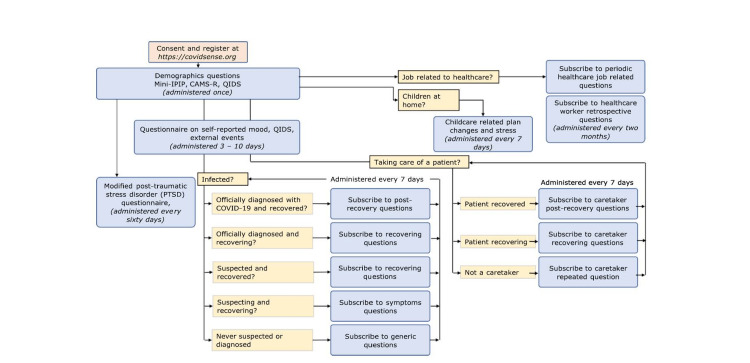
Complete diagram of CovidSense question paths.

The conceptual outline of our study design is shown in Figure 1, and a complete state diagram is shown in Figure 2.

CovidSense works by sending messages to participants at regular intervals of every 10 days. The participants are then redirected to a website where they respond to a question set that is a function of their previous responses. The longitudinal study can be broadly split into two categories: (1) the initiation stage, which is common to all participants, and (2) the periodic question set phase, which is custom-tailored to each participant.

In the initiation stage, participants are inducted into the study by signing up on covidsense.org, where they are required to provide their cellphone numbers. Once registered, the participant gets the first set of questions which asks for basic information, such as location, age, biological sex, profession, yearly income before the pandemic started, and marital status. The second part of the question set is a modified version of the Quick Inventory of Depressive Symptomatology (QIDS) [13]. The third part of the questionnaire is the Cognitive and Affective Mindfulness Scale-Revised (CAMS-R) [14]. In addition to demographics and psychoanalytical questions, the participants are also asked if their profession is related to healthcare.

The periodic stage, the second part of the longitudinal study, involves a periodic set of questions adapted to each participant. Based on the response, the participants are categorized into current patients who contracted the virus, current caretakers who are taking care of a COVID-19 patient, healthcare workers, and the rest of the participants. A common set of questions sent out every 10 days collects information about the participants’ mood, QIDS score, and how they react to external conditions, such as quarantine, news, and city/state-wide lockdown.

Procedures

The responses from the initial questionnaire (panel S) were used for analyzing the demographics of the participants, such as age, gender, geographical location, participant status (patient, caretaker, etc.), and pre-existing conditions. The initial questionnaire was also utilized for extracting the 20-item mindfulness score (CAMS-R) [14] and the 16-item, five big International Personality Item Pool (IPIP) [15]. The CAMS-R scores indicate the ability of an individual to cope with varying external conditions; an individual with a high CAMS-R score is more likely to be resilient to external conditions than an individual with a low score. The IPIP score quantifies the personality traits of a person. Of the five scores, we found neuroticism, which indicated the emotional stability of a person, to be most useful in our analysis. A person with a higher score on neuroticism tended to have more mood swings and was likely to be susceptible to external stressors. In contrast, participants with lower scores are more resilient and less likely to be affected by external conditions. Mindfulness and neuroticism were hypothesized to be correlated to how each group coped during the ongoing pandemic. These observations were utilized to perform a static analysis of the participant categories. The periodic responses were then used to extract the depressive symptomatology scores (QIDS), self-reported mood, self-reported change in substance use, self-reported change in stress levels, and self-reported physical symptoms, including cough, cold, bodyaches, temperature, and gastrointestinal problems. These observations were used to perform a temporal analysis of various participant groups with emphasis on the correlation between various observations.

For quantifying qualitative questions, responses such as CAMS-R, IPIP, and QIDS were explicitly numeric, while the self-reported questions, such as mood, substance use, change in stress levels, and physical symptoms, had qualitative responses. Responses were converted to numerical values.

For aggregating temporal responses, participants registered at different times during the study, implying that periodic responses such as QIDS will not be aligned in time. To effectively align the responses from a diverse set of response times, the time axis was binned into groups of 10 days. The responses within each time period would then contribute to the aggregate response such as mean and standard deviation.

Statistical analysis

Statistical analysis of the data was broadly done in two parts. In the first part, we analyzed the static parameters of various groups, including CAMS-R and IPIP, and correlated them to gender, age group, participant status, and pre-existing conditions.

The QIDS scores of participants were analyzed over three divisions of the study period corresponding to early, mid, and late periods. In the second part, temporal trends of various groups, including QIDS, and self-reported metrics, such as mood, stress, change in substance use, and physical symptoms, were analyzed at a finer time resolution. The variables were then regressed over time intervals of 10 days to ensure that there were sufficient responses within each time frame. All comparisons relied on the mean and standard deviation of the aggregates. The significance of our findings was computed using a Mann-Whitney U parametric test as our distributions were not Gaussian.

## Results

This study is a first-of-its-kind longitudinal study to understand the long-term effects of the COVID-19 pandemic. The study included 1,190 participants from 65 countries who answered a total of 18,783 questionnaires. The demographic split of participants according to various categories is shown in Table 1. A majority of the participants were from the United States, followed by India, South Africa, and the United Kingdom. Various participants came from 59 other countries, with each country contributing fewer than 15 participants. Most of the respondents were never married (55.7%) and were currently employed (80.6%).

**Table 1:** Participant demographics along with CAMS-R, personality measures, and QIDS over three periods (early, mid, and late). Low CAMS-R scores correlated with high neuroticism and high QIDS. The QIDS scores were higher in the July to September period for all categories. Moreover, participants in the 18-25-year age group and those with pre-existing mental or pulmonary health conditions reported higher QIDS scores than others in their corresponding categories. CAMS-R: Cognitive and Affective Mindfulness Scale-Revised; IPIP: International Personality Item Pool; QIDS: Quick Inventory of Depressive Symptomatology; COVID-19: coronavirus disease 2019

	Sample size	CAMS-R	Neuroticism (IPIP)	QIDS (April 1–June 30)	QIDS (July 1–September 30)	QIDS (October 1–December 31)
Total	1,190	25.7 ± 6.2	2.0 ± 0.6	7.0 ± 4.6	8.2 ± 6.0	6.5 ± 5.4
Gender						
Male	741	26.9 ± 6.6	2 ± 0.6	5.2 ± 3.8	6.0 ± 5.5	6.8 ± 5.3
Female	436	25.0 ± 5.9	2 ± 0.5	7.1 ± 4.4	8 ± 5.7	4.2 ± 4.3
Did not answer	8	25.7 ± 3	3.8 ± 0.5	9.9 ± 5.7	6.6 ± 5.9	4.3 ± 1.9
Age group (years)						
18–25	234	24.7 ± 6.3	2.1 ± 0.6	8.2 ± 4.9	10.5 ± 6.3	8.5 ± 6.2
25–40	420	24.9 ± 5.7	2 ± 0.6	7.0 ± 4.3	8.5 ± 6.1	6.6 ± 5.9
40–60	356	27 ± 6	2 ± 0.6	6.5 ± 4.4	7.3 ± 5.3	6.2 ± 5.2
≥60	169	29.8 ± 6.1	1.9 ± 0.6	5.5 ± 3.8	5.4 ± 4.5	5.2 ± 4.4
Participant status						
Healthcare workers	310	27 ± 6	2.0 ± 0.4	7.1 ± 4.6	7.8 ± 5.7	5.7 ± 4.9
COVID-19 patients	91	27.8 ± 6.8	2.0 ± 0.6	9.0 ± 3.6	10.3 ± 4.5	7.3 ± 4.3
Caretakers	50	25.7 ± 6.0	2.0 ± 0.5	8.0 ± 4.0	8.9 ± 6.0	6.0 ± 3.0
Others	739	25.1 ± 6.2	2.0 ± 0.6	6.2 ± 4.3	7.4 ± 5.7	6.0 ± 5.2
Pre-existing conditions						
Cardiovascular	190	26.8 ± 6.8	2.0 ± 0.6	6.9 ± 4.4	7.9 ± 5.6	6.3 ± 5.0
Mental health	491	24.2 ± 5.9	2.1 ± 0.6	8.3 ± 4.7	9.8 ± 6.2	7.9 ± 5.8
Pulmonary	118	25 ± 5.3	2.0 ± 0.6	8.9 ± 5.1	10.6 ± 6.6	10.1 ± 7.2
Other health issues	71	24.6 ± 5.4	2.0 ± 0.6	7.2 ± 4.8	8.7 ± 6.0	6.4 ± 4.7
None	537	27.0 ± 5.9	2.0 ± 0.5	5.6 ± 3.8	5.6 ± 4.6	4.6 ± 4.2
Participant country						
United States	595	26.1 ± 5.5	2.0 ± 0.6	6.6 ± 4.4	6.5 ± 5.2	5.7 ± 5.0
India	223	26.2 ± 6.3	2.0 ± 0.6	7.1 ± 4.7	8.7 ± 5.7	6.6 ± 4.7
South Africa	109	25.3 ± 7.2	2.0 ± 0.5	6.9 ± 5.7	10.6 ± 6.1	8.9 ± 6.0
United Kingdom	65	24.4 ± 5.9	2.0 ± 0.6	3.3 ± 2.2	11.3 ± 7.6	9.7 ± 8.7
Mexico	35	25.2 ± 5.5	2.0 ± 0.6	NA	9.7 ± 5.1	5.2 ± 4.3
Others	163	25.3 ± 6.6	2.0 ± 0.5	6.3 ± 4.0	7.4 ± 4.8	5.53.7

CAMS-R and IPIP analysis

Table 1 shows the mean and standard deviation of CAMS-R and IPIP scores. Participants without any pre-existing conditions reported a higher CAMS-R score compared to participants with pre-existing conditions (p < 0.0001). The neuroticism score for participants with pre-existing mental health was slightly higher (p < 0.02), implying that they were more prone to mood changes. Additionally, participants in the age group of 18-25 years had slightly higher neuroticism scores (p < 0.01). We observed that the higher neuroticism scores had an effect on the depressive scores of patients with pre-existing conditions, as we will see in the later sections.

QIDS analysis

Table 1 shows QIDS scores for various pre-existing conditions divided into three phases. Two groups showed significant variances, namely, participants divided by age, and participants divided by pre-existing conditions.

Participants in the age group of 18-25 years reported higher QIDS scores than others in this category and reported worse moods. Participants with either mental health conditions or pulmonary conditions had worse QIDS scores (p < 0.0001). Over the study period, participants with pre-existing mental health reported moderate-to-severe QIDS scores (≥10) 36.6% of the time, while those with pulmonary conditions reported moderate-to-severe QIDS scores 46% of the time. In contrast, people without any pre-existing conditions reported moderate-to-severe QIDS scores only 13% of the time. We also observed that there was a uniform increase in QIDS scores between July 1 and September 30 (p < 0.0001), corresponding to an overall increase in cases globally.

Correlation between QIDS and self-reported mood

**Figure 3:**
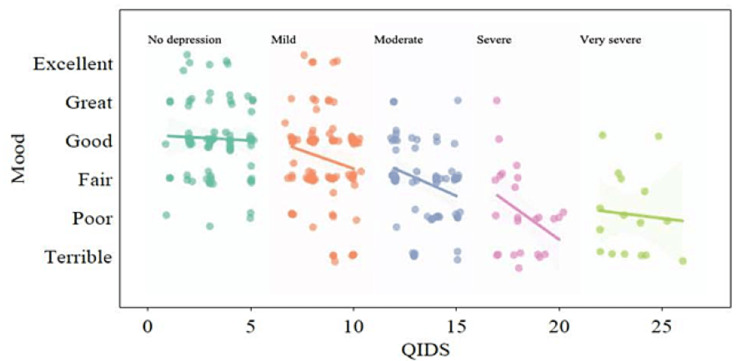
Correlation between self-reported mood and QIDS. The self-reported mood questionnaire correlates well with the QIDS questionnaire, particularly for participants with moderate-to-severe depression scores (QIDS >10). QIDS: Quick Inventory of Depressive Symptomatology

Figure 3 shows a scatter plot of QIDS and self-reported mood, along with regression over various regions of QIDS. We noticed that there was a stronger correlation between mood and QIDS in the moderate and severe depression scores (QIDS between 10 and 20) (p < 0.005). There was a smaller correlation in lower QIDS regions, which is consistent with clinical findings. The relationship between an increase in QIDS score and worsening self-reported mood for various groups is also relevant in the time series, as will be discussed next.

Temporal analysis

**Figure 4:**
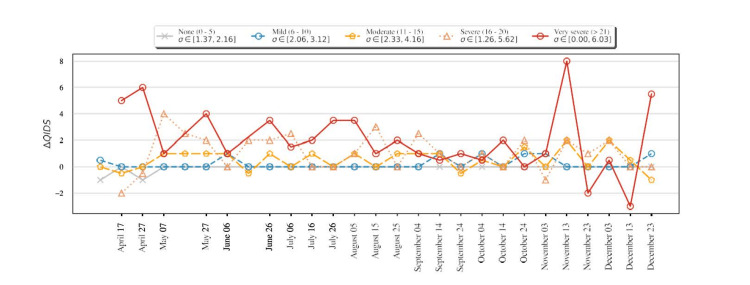
Variation of QIDS for various ranges of QIDS. σ represents the variance of the quantity at each timestamp. We observed that participants with moderate-to-very severe QIDS scores had larger variations in QIDS. QIDS: Quick Inventory of Depressive Symptomatology

**Figure 5:**
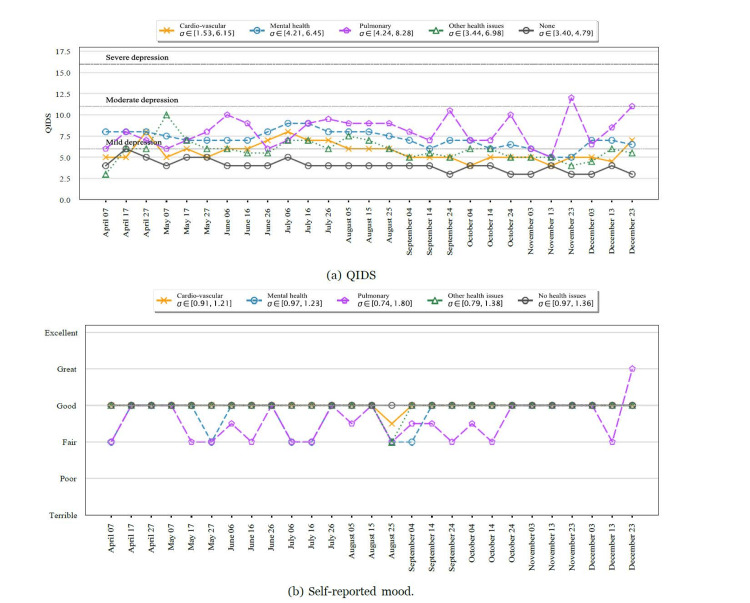
Temporal dynamics of QIDS for participants with pre-existing conditions. σ represents the variance of the quantity at each timestamp. Participants with any pre-existing condition showed higher QIDS scores throughout the study period. QIDS: Quick Inventory of Depressive Symptomatology

**Figure 6:**
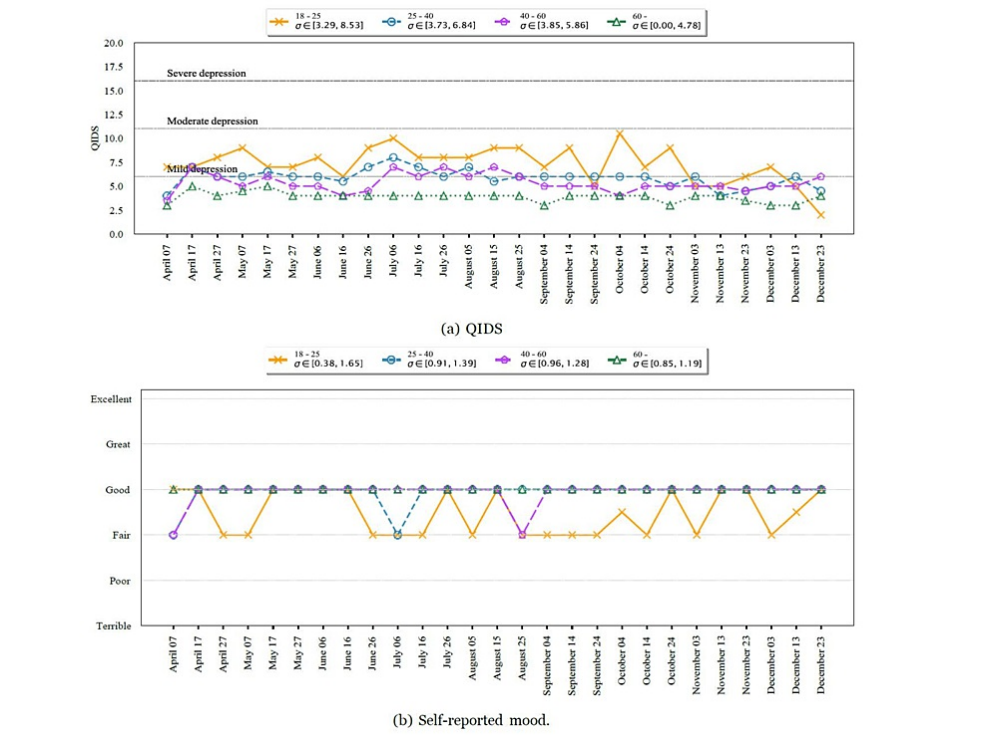
Temporal dynamics of QIDS for various age groups. σ represents the variance of the quantity at each timestamp. Over the study period, participants in the age group of 18-25 years reported higher QIDS scores and worse mood than other age groups. QIDS: Quick Inventory of Depressive Symptomatology

We analyzed the change in QIDS between consecutive responses and segregated them into various categories based on the first of the two QIDS responses. The results are shown in Figure 4. We aggregated and plotted the QIDS scores for participants with various pre-existing conditions in Figure 5, Panel a, and for various age groups in Figure 6, Panel a for the whole study period. We noticed that participants who reported a higher QIDS score were more likely to have larger variations in their consecutive QIDS responses (p < 10^−8^). This was particularly noticeable in the early phase of the study (April to October). We also observed a large increase in QIDS scores for participants reporting very severe scores, possibly due to the presidential election in the United States.

Consistent with the results in the previous section, participants in the age group of 18-25 years and those with pulmonary or mental health conditions in the past reported a larger QIDS score for most time periods. In addition, participants with pulmonary conditions showed larger variations in QIDS score, possibly due to varying levels of infection spread. The self-reported mood score trends were similar to the QIDS score trends, as shown in Figure 5, Panel b, where participants with mental or pulmonary health conditions consistently reported a poorer self-assessment of mood compared to others (p < 0.0001). This finding was consistent for every reporting period of 10 days. Similarly, participants in the age group of 18-25 years reported a poorer self-assessment compared to others, as shown in Figure 6, Panel b.

**Figure 7:**
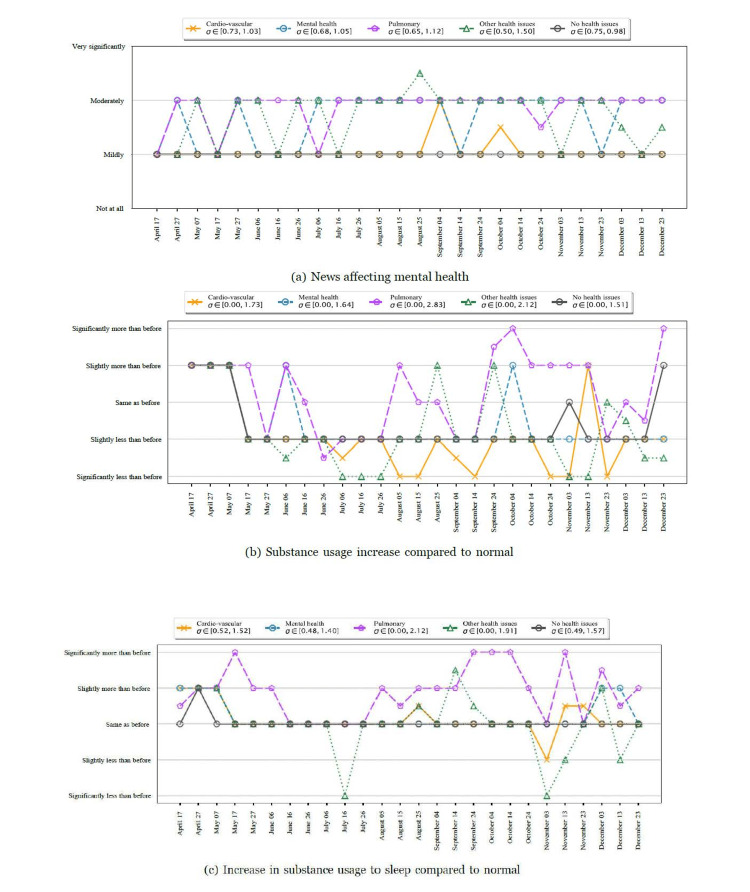
Temporal dynamics of external stressors. σ represents the variance of the quantity at each timestamp. The comparisons are made for participants who reported a change in their substance use. Consistent with our other observations, we found that participants with pulmonary and mental health issues were more severely affected by the news articles, had at least a slight increase in substance use, and required more than normal substance intake to sleep.

Among participants with various pre-existing conditions, we found variability in their response to external events such as news and substance usage. Figure 7a shows the responses to how participants were affected by the news. Participants with no health issues felt that the news affected them moderately or very significantly only 26% of the time. In contrast, participants with pulmonary conditions responded that the news affected them moderately or very significantly 58% of the time, while those with mental health conditions responded the same 52% of the time.

One out of three participants reported changes in substance use at least once during the study period (Figure 7, Panel b). Among these participants, we found a similar trend among participants with various conditions. Participants with pulmonary conditions more often reported an increase in substance use compared to other groups (p < 0.004), and an increase in substance use to sleep (p < 0.0001). We found no significant increase in substance use for participants with pre-existing mental health conditions; however, those participants reported an increase in substance use required to sleep (p < 0.0001).

## Discussion

CovidSense is an adaptive longitudinal study designed to track the fluctuations of mental health issues during the pandemic in the general population. To date, it is the longest longitudinal study on the matter. The main conclusions of the study are as follows: participants in the age group of 18-25 years showed higher neuroticism, lower baseline mindfulness, and overall worse depressive symptoms than their older counterparts. The younger age group is thought to be at a higher risk of mental health conditions based on their CAMS-R and neuroticism scores. This is further supported by their higher QIDS scores over the entire study duration (p < 0.00001) and poorer self-assessment of r own mood. This result is consistent with small sample studies [16-18] highlighting the relationship between increased stress, disrupted daytime routines, and depression in youth. Of note, studies have shown increased distress in children and adolescents, but our study extends those findings to college-aged youth.

Taken together, these findings emphasize the need for both preventive, resilience-promoting, and treatment interventions for this specific age group [19]. While younger individuals were thought to be at lower risk from the virus itself, they proved to be more susceptible to the mental health effects of the pandemic.

Participants with pre-existing conditions were more susceptible to the pandemic and lockdown conditions

This finding was consistent with our initial hypothesis and the literature covering short periods of time and was confirmed by average QIDS scores, as well as temporal dynamics. Among individuals with pre-existing conditions, we found that participants with a history of pulmonary conditions showed increased depressive scores compared to other groups. This result was intuitively logical based on the potentially devastating effects of COVID-19 on the respiratory system [20]. These findings were further confirmed by our analysis of changes in substance use and how the news affected them. Overall, 14.7% of the participants with no pre-existing conditions reported an increase in substance use at least once, while 17.9% of participants with pulmonary conditions and 21.8% of the participants with mental health conditions reported an increase in substance usage at least once. Additionally, 7.8% of the participants with no pre-existing conditions reported that they were moderately or very severely affected by the news at least once. In contrast, 23.5% of the participants with pulmonary conditions and 25.9% of the participants with mental health conditions replied being affected by news moderately or very severely at least once. This is in line with a small survey reporting that individuals with rare diseases consistently had higher rates of depression through the pandemic compared to the general public [21], suggesting a need for widespread screening and early intervention in these groups, which were not necessarily considered high risk prior to the pandemic.

Participants with pre-existing conditions were more sensitive to news

An important finding of our analysis was that news had a variable effect on different groups. Participants with pre-existing mental health conditions and pulmonary conditions were more sensitive to news articles. Much has been written about the pervasive impact of 24/7 connectivity on the human condition, with effects ranging from sleep disturbances to increased anxiety and poorer concentration [22], as well as the potentially detrimental effect of increased news exposure. Our results support that over the course of the pandemic, news had a negative impact on mental health.

Self-reported mood was more consistent with QIDS for participants in the moderate-to-very severe clinical depression conditions

A unique feature of our study was the simultaneous measurement of the QIDS scores, as well as self-reported mood. Our analysis showed that self-reported mood is a reliable indicator of a person’s mental health condition, particularly when the QIDS score pointed to moderate-to-severe depressive symptoms. Thus, while validating electronically self-report mood (using ecological momentary assessment, EMA) as screening for depressive symptoms was not a stated goal of the study, our findings support the potential usefulness of EMA as a wide-ranging screening tool for depressive disorders in the general population. The acceptability of screening by SMS/phone is echoed by a reported increase in the use of digital forums, websites, and other technology-based assistance for mental distress during the pandemic [23].

Study limitations

The CovidSense study shed some light on the effect of the pandemic on various populations but has several limitations related to both the timing and execution that merit discussion. The study was rolled out as fast as possible with the start of the pandemic, with considerable uncertainty in surrounding worldwide circumstances, and therefore lacked a full baseline objective assessment of participants’ physical and mental health; hence, we report only subjective findings. The choice of SMS texts rather than an app download was a conscious decision brought about by the desire to be as inclusive as possible and not exclude individuals with limited smartphone access, yet it is possible that the sample does not cover groups with no/limited phone access. The design of CovidSense, though not without limitations, may explain some differences in the results between our study and some of the early literature documenting mental distress in the pandemic. Specifically, the increased mood and QIDS variability in individuals with pre-existing conditions highlights the intimate relationship between external stressors and mood responses. This could represent a potential reason as to why previous studies failed to establish clear trajectories for mood/anxiety disorders in studied samples as the events of the past 1.5 years have been largely unprecedented for the current human generations.

## Conclusions

Regarding design, the tests in CovidSense were selected based on potential future usefulness; the CAMS-R was selected as mindfulness is thought to have a protective factor considering stress. The QIDS-SR, as a self-report measure, is closely correlated with the Hamilton Depressive Inventory, the gold standard clinician-rated scale for depression, and while it does not replace a clinical interview, it allows a closer assessment of depression prevalence in the surveyed population. We hope that the insights from the temporal analyses provided here will enable future policymaking to be more efficient at directing medical resources to reduce the impact of the COVID-19 pandemic and any future pandemics.
